# Clinical presentation and neurovascular manifestations of cardiac myxomas and papillary fibroelastomas: a retrospective single-institution cohort study

**DOI:** 10.3389/fcvm.2023.1222179

**Published:** 2023-09-01

**Authors:** Akshay Mathavan, Akash Mathavan, Urszula Krekora, Mohit Mathavan, Vanessa Rodriguez, Ellery Altshuler, Brianna Nguyen, Mohammed Ruzieh

**Affiliations:** ^1^ Department of Internal Medicine, University of Florida, Gainesville, FL, United States; ^2^ University of Central Florida College of Medicine, University of Central Florida, Orlando, FL, United States; ^3^ Department of Family Medicine, Ocala Hospital, Ocala, FL, United States; ^4^ Department of Internal Medicine, University of South Florida, Tampa, FL, United States; ^5^ University of Florida College of Medicine, University of Florida, Gainesville, FL, United States; ^6^ Division of Cardiovascular Medicine, University of Florida, Gainesville, FL, United States

**Keywords:** cardiac myxomas, cardiac papillary fibroelastoma, neutrophil–lymphocyte (N/L ratio), cerebrovascular events, benign cardiac neoplasms

## Abstract

**Background:**

Primary cardiac tumors are often benign and commonly present as cardiac myxomas (CMs) or papillary fibroelastomas (CPFEs). There is a paucity of prognostic indicators for tumor burden or potential for embolic cerebrovascular events (CVEs). This study was performed to address these gaps.

**Methods:**

Medical records at the University of Florida Health Shands Hospital between 1996 and 2021 were screened to identify patients with CMs or CPFEs. Clinical features, echocardiographic reports, and CVE outcomes were quantitatively assessed.

**Results:**

A total of 55 patients were included in the study: 28 CM (50.9%) and 27 CPFE (49.1%) patients. Baseline patient characteristics were similar among patients. The neutrophil–lymphocyte ratio was correlated (*p* < 0.005 in all cases) to three metrics of tumor size in both CM (*r *= 64–67%) and CPFE (*r *= 56–59%). CVEs were the presenting symptom in 30 (54.5%) patients. CVE recurrence was high; the 5-year CVE recurrence rate in patients with tumor resection was 24.0% compared to 60.0% without resection. No baseline patient characteristics or tumor features were associated with an initial presentation of CVEs compared to any other indication. Univariate analysis indicated that prolonged duration to surgical resection, left atrial enlargement, male sex, and a neutrophil–lymphocyte ratio >3.0 at the follow-up were significantly associated with 5-year CVE recurrence. Left atrial enlargement and a neutrophil–lymphocyte ratio >3.0 at the follow-up remained significantly associated with 5-year CVE recurrence in multivariate analysis.

**Conclusion:**

The neutrophil–lymphocyte ratio may prognosticate tumor size and recurrence of neurologic events. An increased risk of CVE within 5 years of mass resection is almost exclusive to patients initially presenting with CVEs.

## Introduction

Primary cardiac tumors are rare neoplasms with an annual global incidence of less than 0.2% (1). Approximately 90% of these tumors are benign, the majority of which are cardiac myxomas (CMs) and papillary fibroelastomas (CPFEs). CMs arise from multipotent mesenchymal cells and consist of a myxoid matrix with an acid-mucopolysaccharide-rich stroma (2). Meanwhile, CPFEs are benign lesions derived from valvular endocardium with unknown pathogenesis. There is debate about whether CPFEs represent true neoplasms or reactive tumors; CPFEs are suggested to be hamartomas, neoplasms, or organizing thrombi, in which they originate from aggregates of microthrombi at sites of endothelial damage that evolve into a mass (3). Although generally identified postmortem, increasing utilization and resolution of echocardiographic modalities have shown that CPFEs may have a prevalence equivalent to CMs (4). Clinical presentation for these benign cardiac neoplasms varies and significantly depends on tumor location. Left-sided CMs present with obstructive and constitutional symptoms and systemic embolisms. CPFEs, especially those found on the mitral and aortic valves, are associated with thromboembolic disease (5, 6).

The long-term prognosis of CMs and CPFEs is good, with low recurrence rates often associated with either a relevant family history or an absence of negative margins on surgical excision (1, 7). There is a relative scarcity of understanding of the pathogenesis, clinical profile differences, tumor size markers, and long-term prognosis of CMs and CPFEs. Therefore, this institutional retrospective cohort study aims to provide a comprehensive characterization of the clinical course of patients diagnosed with CMs and CPFEs. In the study group, we emphasize potential prognostic markers, including neutrophil–lymphocyte ratio (NLR), and underline neurovascular manifestations.

## Methods

### Design, database, and study population

A retrospective chart review of adult patients treated at the University of Florida Health Shands Hospital (UFHSH), a large academic tertiary care medical center in Gainesville, FL, USA, was conducted. Patients were identified using the UFHSH integrated data repository. Inclusion criteria for the study included adult patients (age ≥ 18 years) diagnosed with a benign cardiac neoplasm (International Classification of Diseases 9 and 10 codes 212.7 and D15.1, respectively) who were treated at UFHSH between January 1996 and January 2021. The time range was limited by the availability of digitized records prior to 1996. Of this initial pool of patients, only those diagnosed with CMs or CPFEs were included in the final investigation. In all cases in which surgical resection of the tumor was performed, the diagnosis was supported by histopathological confirmation. In the remaining cases in which surgical resection was not performed, the diagnosis was supported by clinical presentation and characteristic features on echocardiographic imaging (8). Baseline demographic data and clinical and pathological information from medical records were also extracted. The study was approved by the University of Florida Institutional Review Board (IRB202102647).

The hematologic laboratory results presented in this study reflect those collected at the time of presentation and at the time of follow-up evaluation. NLR was defined as the ratio between absolute neutrophil and lymphocyte counts. Measurements of tumors in two-dimensional echocardiograms were collected as longer dimension *a* (mm) and shorter dimension *b* (mm). Tumor size was estimated using the arithmetic mean (a×b/2), geometric mean ($\sqrt{a\;\times \;b}$), and area (a×b) of the dimensions.

### Statistical analysis

Nonparametric statistical analysis was performed using SPSS 28.0.1.0 (142) (IBM Corp, Armonk, NY, USA). Continuous variables were compared using the Mann–Whitney *U* test, while categorical variables were compared using Pearson's chi-squared test. Correlations between continuous variables are presented as the Pearson product-moment correlation coefficient *r*. Univariate and multivariate survival analyses were performed with Cox proportional hazards regression models; the proportional hazards assumption was not violated in these analyses. All statistical tests were two-sided, and *p*-values <0.05 were deemed statistically significant.

## Results

### Patient characteristics and presentation

A total of 62 patients met the inclusion criteria during the study period, including 28 (45.2%) patients with CMs and 27 (43.5%) patients with CPFEs. The remaining seven cases were lipomas (Supplementary Figure S1). The characteristics and presentation of patients are reported in Table 1. Baseline demographics did not vary significantly between CM and CPFE groups. The mean age during tumor identification was 56 years, and patients were predominantly women [*n* = 17 (60.7%) CM patients and *n* = 18 (66.7%) CPFE patients]. The most common comorbidities at presentation were hypertension (*n* = 35, 63.6%), hyperlipidemia (*n* = 32, 58.2%), and coronary artery disease (*n* = 24, 43.6%). Compared to patients with CMs, patients with CPFEs were significantly more likely to present with moderate or severe valve stenosis or regurgitation (*n* = 1, 3.6% vs. *n* = 6, 22.2%; *p* = 0.038) or have had prior cardiac surgery (*n* = 1, 3.6% vs. *n* = 8, 29.6%; *p* = 0.009).

**Table 1:** Characteristics and presentation of patients with benign cardiac neoplasms.

Parameter	Benign cardiac neoplasm		*P*-value
	CM (*n* = 28)	CPFE (*n* = 27)	
Age (years)	53.7 ± 15.3	58.5 ± 15.8	0.505
Sex			
Male	11 (39.3%)	9 (33.3%)	0.646
Female	17 (60.7%)	18 (66.7%)	
Race			
Caucasian	27 (96.4%)	24 (88.9%)	0.282
Black/African-American	1 (3.6%)	2 (7.4%)	
Other	0 (0%)	1 (3.7%)	
BMI (kg/m^2^)	26.9 ± 4.5	27.7 ± 6.2	0.585
BSA (m^2^)	1.9 ± 0.2	1.9 ± 0.3	0.989
Comorbidities			
Hypertension	15 (53.6%)	20 (74.1%)	0.114
Hyperlipidemia	13 (46.4%)	19 (70.4%)	0.072
Coronary artery disease	11 (39.3%)	13 (48.1%)	0.508
Diabetes mellitus	4 (14.3%)	5 (18.5%)	0.671
Chronic kidney disease	2 (7.1%)	4 (14.8%)	0.362
Atrial fibrillation	3 (10.7%)	2 (7.4%)	0.670
Valvular heart disease	1 (3.6%)	6 (22.2%)	**0**.**038**
Aortic stenosis	1 (100%)	4 (66.7%)	
Bicuspid aortic valve	0 (0%)	1 (16.7%)	
Mitral stenosis	0 (0%)	1 (16.7%)	
Mitral regurgitation	0 (0%)	1 (16.7%)	
Mitral valve prolapse	0 (0%)	1 (16.7%)	
Prior CVE	7 (25.0%)	5 (18.5%)	0.561
Prior cardiac surgery	1 (3.6%)	8 (29.6%)	**0**.**009**
History of smoking	15 (53.6%)	12 (44.4%)	0.499
History of IVDU	5 (17.9%)	1 (3.7%)	0.092
Primary symptom			
Cerebrovascular event	17 (60.7%)	13 (48.1%)	0.349
Chest pain	3 (10.7%)	8 (29.6%)	
Dyspnea	4 (14.3%)	5 (18.5%)	
Nonspecific	3 (10.7%)	0 (0%)	
Incidental	1 (3.6%)	1 (3.7%)	
Presence of murmur	6 (21.4%)	1 (3.7%)	**0**.**049**
Hematologic labs			
HGB (g/dl)	12.1 ± 1.8	12.3 ± 1.8	0.682
PLT (×10^9^/L)	256.7 ± 102.3	225.9 ± 84.3	0.230
WBC count (×10^9^/L)	13.9 ± 6.2	10.4 ± 4.6	**0**.**021**
NLR	8.1 ± 3.9	4.4 ± 1.7	**<0**.**001**

Bolded *p*-values indicate statistically significant results.

Age, BMI, BSA, and hematologic values are reported as average ± one standard deviation. Composition of valvular heart disease is reported as the number of patients (% of patients with valvular dysfunction). All other values are reported as the number of patients (% of the total number of patients). History of smoking is defined as greater than 10 pack-years. Nonspecific symptoms include constitutional symptoms such as weight loss, fatigue, and night sweats.

BMI, body mass index; BSA, body surface area; CM, cardiac myxoma; CPFE, cardiac papillary fibroelastoma; CVE, cerebrovascular event; HGB, hemoglobin; IVDU, intravenous drug use; NLR, neutrophil–lymphocyte ratio; PLT, platelet; WBC, white blood cell.

The most common presenting symptoms to the hospital were those related to CVE (*n* = 30, 54.5%), chest pain (*n* = 11, 20%), and dyspnea (*n* = 9, 16.4%). In the 30 patients initially presenting with CVEs, 17 (56.7%) patients had CMs, and 13 (43.3%) patients had CPFEs (Supplementary Table S1). Ischemic embolic stroke occurred in 18 patients (10 in the CM and eight in the CPFE group), while 12 patients (seven in the CM and five in the CPFE group) had transient ischemic attacks. No patient presented with primary intracerebral or subarachnoid hemorrhage. In patients with ischemic embolic stroke, the presentation had a wide range of severity with a National Institutes of Health Stroke Scale (NIHSS) score of 5–14. The NIHSS score on presentation was significantly higher in patients with CMs than in patients with CPFEs (8.9 ± 2.3 vs. 5.8 ± 3.8; *p* = 0.046).

The white blood cell count was elevated but significantly more elevated in patients with CMs than in patients with CPFEs (13.9 ± 6.2 vs. 10.4 ± 4.6 × 10^9^/l; *p* = 0.021). NLR was similarly elevated in both groups but significantly more elevated in patients with CMs than in patients with CPFEs (8.1 ± 3.9 vs. 4.4 ± 1.7; *p* < 0.001). Hemoglobin and platelet levels were similar between both groups. The significance of elevations in the white blood cell count and NLR did not vary between the indication for patient presentation (*p* > 0.05 in all cases).

### Echocardiographic results

The results of echocardiograms performed during the initial patient evaluation are presented in Table 2. Of note, 36 (65.5%) benign cardiac neoplasms were initially detected by transthoracic echocardiograms, while the remaining were identified with subsequent transesophageal echocardiograms. The location of CMs was exclusively nonvalvular, with 20 (71.4%) tumors found in the left atrium and four (14.3%) in the right atrium. Multichamber involvement was seen in two patients; one patient had CMs in the left atrium and left ventricle, and another had CMs in the left and right atria. The location of CPFEs was predominantly valvular, with 19 (70.4%) cases on the aortic valve and five (18.5%) on the mitral valve. Multiple tumors were seen in two patients, where both had one mass on the aortic valve and a secondary mass on the left ventricular wall.

**Table 2:** Echocardiographic results in patients with benign cardiac neoplasms.

Parameter	Benign cardiac neoplasm		*P*-value
	CM (*n* = 28)	CPFE (*n* = 27)	
Indication for echocardiogram			
Exclude Cardioembolism	17 (60.7%)	13 (48.1%)	0.349
Chest pain or CAD	5 (17.9%)	11 (40.7%)	
HF symptoms	1 (3.6%)	2 (7.4%)	
Other	5 (17.9%)	1 (3.7%)	
Ejection fraction (%)			
≤40	1 (3.6%)	4 (14.8%)	0.208
41–49	2 (7.1%)	1 (3.7%)	
≥50	26 (92.9%)	22 (81.5%)	
Left atrial enlargement	11 (39.3%)	7 (25.9%)	0.291
Valve dysfunction	7 (25.0%)	13 (48.1%)	0.074
Pulmonary hypertension (mPAP > 20 mmHg)	10 (35.7%)	8 (29.6%)	0.631
Tumor location			
Aortic valve	0 (0%)	19 (70.4%)	**<0**.**001**
Mitral valve	0 (0%)	5 (18.5%)	
Left atrium	20 (71.4%)	0 (0%)	
Right atrium	4 (14.3%)	0 (0%)	
Left ventricle	2 (7.1%)	1 (3.7%)	
Multi-chamber/valvular	2 (7.1%)	2 (7.4%)	
Tumor size (mm)			
Longer dimension	33.4 ± 9.1	7.7 ± 2.6	**<0**.**001**
Shorter dimension	24.9 ± 7.0	5.6 ± 2.4	**<0**.**001**
Independent mobility	20 (71.4%)	21 (77.8%)	0.589

Bolded *p*-values indicate statistically significant results.

Tumor size is reported as average ± one standard deviation. All other values are reported as the number of patients (% of the total number of patients). Left atrial enlargement is defined as left atrial diameter >40 mm. Valve dysfunction on echocardiogram is defined as the presence of moderate or severe valve stenosis or regurgitation; the severity of dysfunction is assessed in accordance with established guidelines (9, 10).

CAD, coronary artery disease; CM, cardiac myxoma; CPFE, cardiac papillary fibroelastoma; HF, heart failure; mPAP, mean pulmonary artery pressure.

Compared to those in CPFEs, measurements of tumor size in CMs were significantly larger for dimensions *a* (33.4 ± 9.1 vs. 7.7 ± 2.6; *p* < 0.001) and *b* (24.9 ± 7.0 vs. 5.6 ± 2.4; *p* < 0.001). In the CM group, NLR was significantly correlated with the arithmetic mean [correlation coefficient *r*(26) = 0.64; *p* < 0.001], geometric mean [*r*(26) = 0.65; *p* < 0.001], and area [*r*(26) = 0.67; *p* < 0.001] of the dimensions of the tumor. In the CPFE group, NLR was also significantly correlated with the tumor's size arithmetic mean [correlation coefficient *r*(25) = 0.59; *p* = 0.001], geometric mean [*r*(25) = 0.56; *p* = 0.002], and area [*r*(25) = 0.59; *p* = 0.001]. The NLR per unit size of tumor (cm), defined as the ratio of NLR to the arithmetic mean of tumor dimensions *a* and *b*, was significantly higher in patients with CPFEs than in patients with CMs (6.9 ± 2.8 vs. 2.7 ± 1.1; *p* < 0.001).

### Surgical and postsurgical outcomes

A total of 48 (87.3%) patients had surgical resection of the tumor, as reported in Table 3. Surgical resection was more likely to be performed in patients with CMs than in patients with CPFEs (*n* = 27, 96.4% vs. *n* = 21, 77.8%; *p* = 0.038). No patients experienced ischemic or hemorrhagic stroke during or within 30 days of surgical resection, and 30-day survival was 100%.

**Table 3:** Surgical and postsurgical outcomes in patients with benign cardiac neoplasms.

Parameter	Benign cardiac neoplasm		*P*-value
	CM (*n* = 28)	CPFE (*n* = 27)	
Surgical resection	27 (96.4%)	21 (77.8%)	**0**.**038**
Concomitant procedure			
CABG	11 (39.3%)	11 (40.7%)	0.912
Valvuloplasty	2 (7.1%)	10 (37.0%)	**0**.**007**
Duration until resection (days)	48.3 ± 35.7	62.3 ± 77.5	0.409
Post-operative complications (*n* = 48; CM: 27 and CPFE: 21)			
Infection	4 (14.8%)	4 (19.0%)	0.581
Tachyarrhythmia	5 (18.5%)	3 (14.3%)	
Bradyarrhythmia	4 (14.8%)	2 (9.5%)	
Bleeding	1 (3.7%)	2 (9.5%)	
Renal insufficiency	1 (3.7%)	2 (9.5%)	
Follow-up period (months)	71.3 ± 16.8	65.7 ± 11.1	0.150
Present at 1 year	28 (100%)	26 (96.3%)	0.303
Present at 5 years	26 (92.9%)	24 (88.9%)	0.610
Hematologic labs at follow-up (*n* = 41; CM: 20 and CPFE: 21)			
WBC count (×10^9^/L)	7.22 ± 2.61	7.55 ± 2.36	0.673
NLR	2.98 ± 1.51	2.96 ± 2.09	0.972
	CVE subset		
	CM (*n* = 17)	CPFE (*n* = 13)	
Meds after discharge			
Aspirin only	11 (64.7%)	8 (61.5%)	0.857
DAPT	4 (23.5%)	2 (15.4%)	
Anticoagulant	2 (11.8%)	3 (23.1%)	
Residual deficit at 1 year	4 (23.5%)	2 (15.4%)	0.582
CVE recurrence			
By 1 year	2 (11.8%)	2 (15.4%)	0.401
By 5 years	5 (29.4%)	4 (30.8%)	0.881
Surgical resection	5 (100%)	1 (25%)	0.048

Bolded *p*-values indicate statistically significant results.

Duration refers to the time between symptom onset and surgical resection of the tumor and is reported as average ± one standard deviation. Postoperative complications are reported as the number of patients (% of patients who underwent surgery). Surgical resection is reported as the number of patients (% of patients with CVE recurrence at 5 years). All other values are reported as the number of patients (% of the total number of patients). The CVE subset refers to the group of patients with benign cardiac neoplasms who initially presented with CVEs.

CABG, coronary artery bypass grafting; CM, cardiac myxoma; CPFE, cardiac papillary fibroelastoma; CVE, cerebrovascular event; DAPT, dual antiplatelet therapy; NLR, neutrophil–lymphocyte ratio; WBC, white blood cell.

The mean follow-up period was 69.3 ± 13.1 months, with 54 (98.2%) patients having at least 1 year of follow-up and 50 (90.9%) patients having at least 5 years of follow-up. Compared to the initial presentation, the mean white blood cell count was significantly lower at the follow-up in both the CM group (difference: −6.68 ± 6.73 × 10^9^/l; *p* < 0.001) and the CPFE group (difference: −2.85 ± 5.17 × 10^9^/l; *p* = 0.013). Compared to the initial presentation, mean NLR was also significantly lower at the follow-up in both the CM group (difference: −5.12 ± 4.18; *p* < 0.001) and the CPFE group (difference: −1.44 ± 2.69; *p* = 0.012). Hematologic values during this follow-up interval did not significantly differ between tumor types (*p* > 0.05 in all cases).

**Figure 1:**
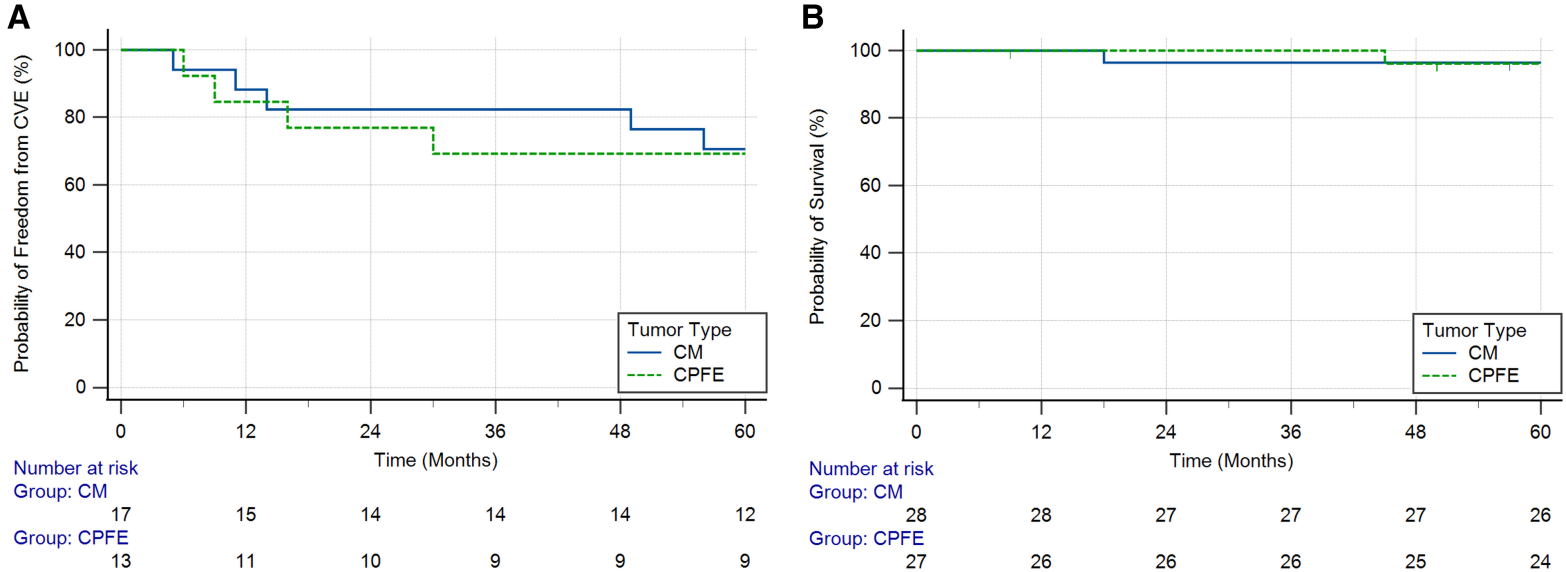
Kaplan–Meier curves showing (**A**) probability of freedom from cerebrovascular events (CVEs) over time in patients with cardiac myxomas (CMs) and cardiac papillary fibroelastomas (CPFEs) who initially presented with CVEs (log-rank test *p* = 0.891) and (**B**) probability of survival over time in patients with CMs and CPFEs (log-rank test *p* = 0.966).

In the subset of 30 patients with benign cardiac neoplasms who initially presented with CVEs (Table 3 and Supplementary Table S2), four (13.3%) had CVE recurrence within 1 year of diagnosis (*n* = 2, 11.8% in the CM group, and *n* = 2, 15.4% in the CPFE group) and nine (30.0%) had recurrence at 5 years (*n* = 5, 29.4% in the CM group and *n* = 4, 30.8% in the CPFE group) (Figure 1A). Among the nine patients with 5-year CVE recurrence, three did not have surgical resection at the time of initial presentation. The 5-year CVE recurrence rate in patients with tumor resection was 24.0% (*n* = 6) compared to 60.0% (*n* = 3) in those without resection (*p* = 0.109). Additionally, three patients experienced other embolic events within 5 years of resection/discharge, including embolization to bilateral popliteal arteries, inferior mesenteric arteries, and left renal artery. One patient who did not initially present with CVEs experienced an ischemic stroke within 5 years of resection of his CM.

Univariate analysis of the subset of patients initially presenting with CVEs indicated that factors significantly associated with CVE recurrence within 5 years included those with a duration between symptom onset and surgical resection >60 days (HR: 4.182; 95% CI: 1.035–16.899; *p* = 0.046), left atrial enlargement (left atrial diameter >40 mm) on the initial echocardiographic report (HR: 8.648; 95% CI: 1.781–41.992; *p* = 0.008), male sex (HR: 5.422; 95% CI: 1.343–21.889; *p* = 0.018), and NLR > 3.0 at the follow-up (HR: 4.173; 95% CI: 1.115–15.618; *p* = 0.034), as reported in Supplementary Table S3. When controlling for age and risk factors for CVEs (i.e., hypertension, hyperlipidemia, coronary artery disease, diabetes mellitus, or coronary artery bypass grafting) in multivariate analysis, left atrial enlargement (HR: 15.932; 95% CI: 1.940–139.142; *p* = 0.013) and NLR > 3.0 at the follow-up (HR: 5.017; 95% CI: 1.480–34.647; *p* = 0.043) remained significantly associated with CVE recurrence within 5 years. The observed trends in these analyses were maintained when stratifying into CM and CPFE tumor types.

Two patients died during the 5-year follow-up period (Figure 1B). One patient with a CM presented 18 months after surgical resection died from severe intraparenchymal hemorrhage, and the second patient with a CPFE presented 45 months after surgical resection died from end-stage heart failure. Tumor recurrence was seen in one patient, who presented with a CM 4 years after the initial resection. This patient had a significant family history of atrial CMs.

## Discussion

In this study, we present clinical, echocardiographic, and laboratory data of 55 patients with benign cardiac tumors—28 patients with CMs and 27 patients with CPFEs (1, 11–13). Similar to prior investigations, most patients in this study presented with embolic phenomena (54.5%) (11, 14–17). Patients were in their fifth decade of life, and about two-thirds were women. We found NLR to correlate with tumor size for both CM and CPFE and predict CVE recurrence. To the best of our knowledge, these are novel findings. Our study showed that patients with CPFEs were more likely to have valvular dysfunction (moderate or severe valve stenosis or regurgitation as assessed in accordance with established guidelines) or a history of cardiac surgery (9, 10). This may lend credence to the theory that endothelial and valvular damage promotes a nidus for microthrombi collection that raises the risk for CPFE. Two reports have also associated CPFEs and β-thalassemia blood disorders, suggesting a similar hemodynamic mechanism (18, 19). However, a recent study investigating the molecular profile of 14 CPFE patients demonstrated mutations of the *KRAS* oncogene in 11 patients, supporting the neoplastic nature of these lesions (20). Based on echocardiographic reports in this study, CMs were often left-sided (85.7%) and most commonly in the left atrium (71.4%). CPFEs were exclusively left-sided, predominantly valvular (92.6%), and most commonly on the aortic valve (70.4%). These findings correlate with the existing literature (4, 11, 17, 21–23).

No specific biomarkers for benign or malignant cardiac neoplasms are currently validated. Leukocytosis is a nonspecific indicator of inflammation. NLR is a readily accessible adjunct prognostic biomarker of growing contemporary interest. It reflects a shift from adaptive to innate immunity due to a combination of disordered immunity and contributions from disease-specific etiologies. The normal range of NLR is typically between 1 and 2, while values higher than 3.0 and lower than 0.7 are considered pathological. Elevated NLR has been demonstrated as a prognostic marker for morbidity and mortality in various chronic diseases (e.g., atherosclerotic disease, cerebrovascular disease) and cancers (e.g., melanoma, breast cancer) (24). In the case of malignancy, neutrophilia is an observed feature of cancer-elicited chronic inflammation, and it is often accompanied by relative lymphocytopenia due to tumor-driven suppression of the cell-mediated immune response (25, 26). NLR has been associated with tumor size, tumor stage, and metastatic potential and has been shown to predict cancer-specific survival, including progression-free and disease-free survival (27).

In this study, the white blood cell count and NLR were elevated in patients presenting with CMs and CPFEs, although values were significantly higher in the former. This is likely due to the substantially larger tumor sizes of CMs than those of CPFEs. It may also naturally reflect the immunologic nature of CMs, which has been described to overproduce interleukin-6 and other various growth factors and cytokines that generate observed constitutional symptoms. Indeed, interleukin-6 has been posited as a marker of recurrence for CMs (28). In CPFEs, the elevated NLR may be a product of endothelial cell activation and chronic valvular inflammation in addition to neoplastic processes (29). Elevated NLR has previously been observed in patients with CMs, and it has been correlated with tumor size (30). NLR values were similarly correlated with various metrics of tumor size in this study (64%–67% in the CM and 56%–59% in the CPFE group), suggesting a role for this biomarker in tumor burden prognostication. Of note, NLR values may also be elevated in ischemic chest pain, CVE, and other stress states present in these patients during hospitalization; however, NLR values were not significantly altered when stratified by the patient presentation (31). Although CMs and CPFEs generally differ in fundamental pathophysiology, there is considerable overlap in the initial clinical manifestation. Moreover, unusual presentations of these tumors (e.g., the deranged echocardiographic appearance of CMs or nonvalvular site of CPFEs) obscure identification (32, 33). Therefore, the patterns of NLR elevation observed in this study may be useful in the setting of a patient in whom a primary cardiac neoplasm is detected, but the exact etiology is unclear.

Importantly, a sizeable portion of patients with either neoplasm (30%–60%) initially present with CVEs, consistent with the results of this investigation. CM-related CVEs have been associated with a friable or villous tumor surface, younger male patients, and tumors with size ≤4.5 cm (14, 15, 34). CPFE-related CVEs have been associated with small tumor size at diagnosis and independent mobility (11, 17). Other echocardiographic characteristics, aortic valve location, or tumor growth rate have not been identified as risk factors (35). In this study, tumors were expectedly left-sided. No baseline patient characteristics, including age, sex, or cardiovascular risk factors such as a history of hypertension, hyperlipidemia, coronary artery disease, diabetes mellitus, smoking, or prior CVE, were significantly associated with an initial cerebrovascular manifestation of the benign cardiac neoplasm compared to any other indication for presentation. The size or independent mobility of the tumor and left atrial enlargement also did not significantly differ between patients initially presenting with CVEs and any other indications.

Elevated recurrence of CVE, compared to baseline age- and sex-matched controls, after excision has also been reported in both CMs and CPFEs. Prolonged duration until surgical excision in patients with CMs and unremoved lesions in patients with CPFEs are associated with CVE recurrence (4, 11, 16). The CVE recurrence rate in our cohort was high: 30.0% at 5 years (24.0% in patients with prior tumor resection and 60.0% in patients without prior resection). The prolonged duration between symptom onset and surgical resection (>60 days), left atrial enlargement, male sex, and NLR > 3.0 at approximately 1-year follow-up were significantly associated with CVE recurrence within 5 years of surgical resection or discharge. When controlling for age and known risk factors for CVE in multivariable analysis, left atrial enlargement and NLR > 3.0 at approximately 1-year follow-up remained significantly associated with 5-year CVE recurrence. Ultimately, these findings may emphasize the need for timely excision of CMs and CPFEs in suitable candidates and the use of NLR as a prognostic biomarker. Importantly, no cases of hemorrhagic transformation intraoperatively or postoperatively have been reported, lessening concern for postischemic stroke patients on bridge therapy.

This investigation has several limitations that must be emphasized. The study design is retrospective and small in sample size, limiting the robustness of the inferences that can be drawn. Our tertiary care center may also be subject to referral bias, possibly explaining the relatively low rates of incidental discovery of these benign cardiac neoplasms compared to other studies. Regarding NLR, the prognostic biomarker is an accessible and cost-effective measure of dysregulated inflammation that has garnered significant contemporary interest in various fields. In numerous large-cohort studies and meta-analyses within the cardiovascular domain, NLR has been significantly associated with the onset of atrial fibrillation, acute coronary syndrome (including correlation with the Global Registry of Acute Coronary Events risk score and SYNTAX score), and acute decompensated heart failure (36–40). However, its use in cardiovascular tumors has not been well characterized, and further large-scale investigations are required to validate the observations within our study. The reference cutoff and the subsequent interpretive potential of NLR may also vary by age, sex, and other factors, although this requires further research (41).

Furthermore, NLR may be elevated in various acute disease states; therefore, its utility may primarily be as an adjunct prognostic tool (after further validation) and not in a diagnostic capacity. A presentation of ischemic stroke is among the acute etiologies associated with elevated NLR (42). Therefore, it would be of great interest to evaluate NLR in patients presenting with CVEs and underlying benign cardiac neoplasms compared to values in patients with CVEs and no malignancy to further validate this work. Finally, this study included a small number of patients in whom the benign cardiac neoplasm was not excised. In these cases, the diagnosis was formulated via clinical presentation and the presence of characteristic echocardiographic features of the tumor (8). A few contemporary studies have discussed the viability of a conservative approach to asymptomatic benign cardiac neoplasms (especially small, incidentally discovered tumors), one that favors antithrombotic medical therapy as opposed to surgical intervention (16, 17, 43, 44). While our investigation failed to highlight a significant elevation in adverse outcomes in patients in whom surgical excision was not pursued, likely due to the small sample size, we feel it is important to include these cases to reflect these considerations; we bear in mind that without histopathological confirmation of tumors in these cases, the uncertainty and risk of misdiagnosis remains.

In conclusion, our study demonstrated a high CVE recurrence rate in patients with benign cardiac tumors and indicates that NLR may serve as a valid adjunct prognostic tool for tumor burden and recurrence of neurologic events.

## Data Availability

The original contributions presented in the study are included in the article/**Supplementary Material**; further inquiries can be directed to the corresponding author.
