# Supplementary material for: Clinical presentation and neurovascular manifestations of cardiac myxomas and papillary fibroelastomas: a retrospective single-institution cohort study
Source: Front Cardiovasc Med. 2023 Sep 1;10:1222179. doi: 10.3389/fcvm.2023.1222179 (PMC10503431; doi:10.3389/fcvm.2023.1222179)
Supplement: Supplementary file 1 [file Datasheet1.docx]

**Supplementary Material for Clinical Presentation and Neurovascular Manifestations of Cardiac Myxomas and Papillary Fibroelastomas: A Retrospective Single-Institution Cohort Study**

**Table S1.** Characterization of neurovascular events in patients with benign cardiac neoplasm.

| **Parameter** | **Benign Cardiac Neoplasm** | | **P-Value** |
| --- | --- | --- | --- |
|  | **CM (n= 17)** | **CPFE (n= 13)** |  |
| Cerebrovascular Event | | | |
| Ischemic Stroke | 10 (58.8%) | 8 (61.5%) | 0.880 |
| TIA | 7 (41.2%) | 5 (38.5%) |  |
| Secondary Symptoms | | | |
| Chest Pain | 6 (35.3%) | 4 (30.8%) | 0.794 |
| Palpitations | 3 (17.6%) | 2 (15.4%) |  |
| Dyspnea | 1 (5.9%) | 2 (15.4%) |  |
| None | 7 (41.2%) | 5 (38.5%) |  |
| NIHSS Score | 8.9 ± 2.3 | 5.8 ± 3.8 | **0.046** |
| tPA Given | 6 (60.0%) | 4 (50.0%) | 0.671 |
| Infarct Chronicity | | | |
| Acute | 6 (35.3%) | 5 (38.5%) | 0.858 |
| Subacute/Chronic | 3 (17.6%) | 3 (23.1%) |  |
| Both | 2 (11.8%) | 2 (15.4%) |  |
| No Infarcts | 6 (35.3%) | 3 (23.1%) |  |
| Infarct Distribution | | | |
| Single | 3 (17.6%) | 4 (30.8%) | 0.399 |
| Multiple | 8 (47.1%) | 6 (46.2%) |  |
| No Infarcts | 6 (35.3%) | 3 (23.1%) |  |

NIHSS score reported as average ± one standard deviation. tPA given reported in number of patients (% of patients who experienced ischemic stroke). All other values reported in number of patients (% of total number of patients). Bolded p-values indicate statistically significant results.

CM= cardiac myxoma; CPFE= cardiac papillary fibroelastoma; CVE= cerebrovascular event; NIHS = National Institutes of Health Stroke Scale; TIA= transient ischemic attack; tPA= tissue plasminogen activator.

**Table S2.** Comparison of select features in patients with and without cerebrovascular events and underlying benign cardiac neoplasm.

| **Parameter** | **Patients with Benign Cardiac Neoplasm** | | **P-Value** |
| --- | --- | --- | --- |
|  | **With CVE (n = 30)** | **Without CVE (n = 25)** |  |
| Age (years) | 54.4 ± 16.7 | 60.6 ± 13.0 | 0.136 |
| Sex | | | |
| Male | 9 (30.0%) | 11 (44.0%) | 0.285 |
| Female | 21 (70.0%) | 14 (56.0%) |  |
| CV Risk Factors | 18 (60.0%) | 18 (72.0%) | 0.352 |
| WBC (×10^9^/L) | 10.8 ± 5.8 | 13.8 ± 5.3 | 0.052 |
| NLR | 6.5 ± 4.3 | 6.0 ± 2.8 | 0.619 |
| Left Atrial Enlargement | 11 (36.7%) | 7 (28.0%) | 0.497 |
| Tumor Type | | | |
| CM | 17 (56.7%) | 11 (44.0%) | 0.347 |
| CPFE | 13 (43.3%) | 14 (56.0%) |  |
| Tumor Location | | | |
| Aortic Valve | 10 (33.3%) | 9 (36.0%) | 0.238 |
| Mitral Valve | 2 (6.7%) | 3 (12.0%) |  |
| Left Atrium | 13 (43.3%) | 7 (28.0%) |  |
| Right Atrium | 1 (3.3%) | 3 (12.0%) |  |
| Left Ventricle | 2 (6.7%) | 1 (4.0%) |  |
| Multi-chamber | 2 (6.7%) | 2 (8.0%) |  |
| Tumor size, area (mm^2^) | | | |
| CM | 894.5 ± 476.3 | 855.5 ± 398.3 | 0.746 |
| CPFE | 53.0 ± 39.0 | 42.0 ± 25.9 | 0.234 |
| Independent Mobility | 22 (73.3%) | 19 (76.0%) | 0.818 |
| Surgical Resection | 25 (83.3%) | 23 (92.0%) | 0.337 |

Age, tumor size, NLR, and WBC reported as average ± one standard deviation. All other values reported in number of patients (% of total number of patients). CV risk factors included the presence of hypertension, hyperlipidemia, coronary artery disease, diabetes mellitus, smoking, or prior CVE. Bolded p-values indicate statistically significant results.

CM= cardiac myxoma; CPFE= cardiac papillary fibroelastoma; CV= cardiovascular; CVE= cerebrovascular event; NLR= neutrophil-lymphocyte ratio; WBC= white blood cell count.

**Table S3.** Association between select factors and five-year CVE recurrence in patients with benign cardiac neoplasm who initially presented with CVE using univariate survival analysis.

| **Parameter** | **Hazard Ratio (95% Confidence Interval)** | **P-Value** |
| --- | --- | --- |
| BMI≥ 30 kg/m^2^ | 0.852 (0.229—3.176) | 0.812 |
| Duration> 60 Days | 4.182 (1.035—16.899) | **0.046** |
| Independent Mobility | 0.756 (0.189—3.027) | 0.693 |
| Left Atrial Enlargement | 8.648 (1.781—41.992) | **0.008** |
| Left-Sided Tumor Location | 1.868 (0.236—14.950) | 0.556 |
| Male Sex | 5.422 (1.343—21.889) | **0.018** |
| No Surgical Resection at Initial Presentation | 3.729 (0.917—15.171) | 0.066 |
| Tumor Area (cm^2^) < 25^th^ Percentile | 0.994 (0.391—3.316) | 0.917 |
| Tumor Area (cm^2^) > 75^th^ Percentile | 1.042 (0.260—4.172) | 0.953 |
| Hematologic Labs on Presentation | | |
| WBC> 10.0×10^9^/L | 1.400 (0.375—5.222) | 0.617 |
| NLR> 6.0 | 1.927 (0.516—7.198) | 0.330 |
| Hematologic Labs at Follow-up (n= 21; Recurrence: 7 and No Recurrence: 14) | | |
| WBC> 10.0×10^9^/L | 4.368 (0.894—21.355) | 0.068 |
| NLR> 3.0 | 4.173 (1.115—15.618) | **0.034** |

Univariate analysis performed using Cox proportional hazards regression. Duration refers to the time between symptom onset and surgical resection of tumor. Left atrial enlargement is defined as left atrial diameter> 40 mm. Left-sided tumor location includes aortic valve, mitral valve, and left atrium. Bolded p-values indicate statistically significant results.

BMI= body mass index; CM= cardiac myxoma; CPFE= cardiac papillary fibroelastoma; CVE= cerebrovascular event; NLR= neutrophil-lymphocyte ratio; WBC= white blood cell count.


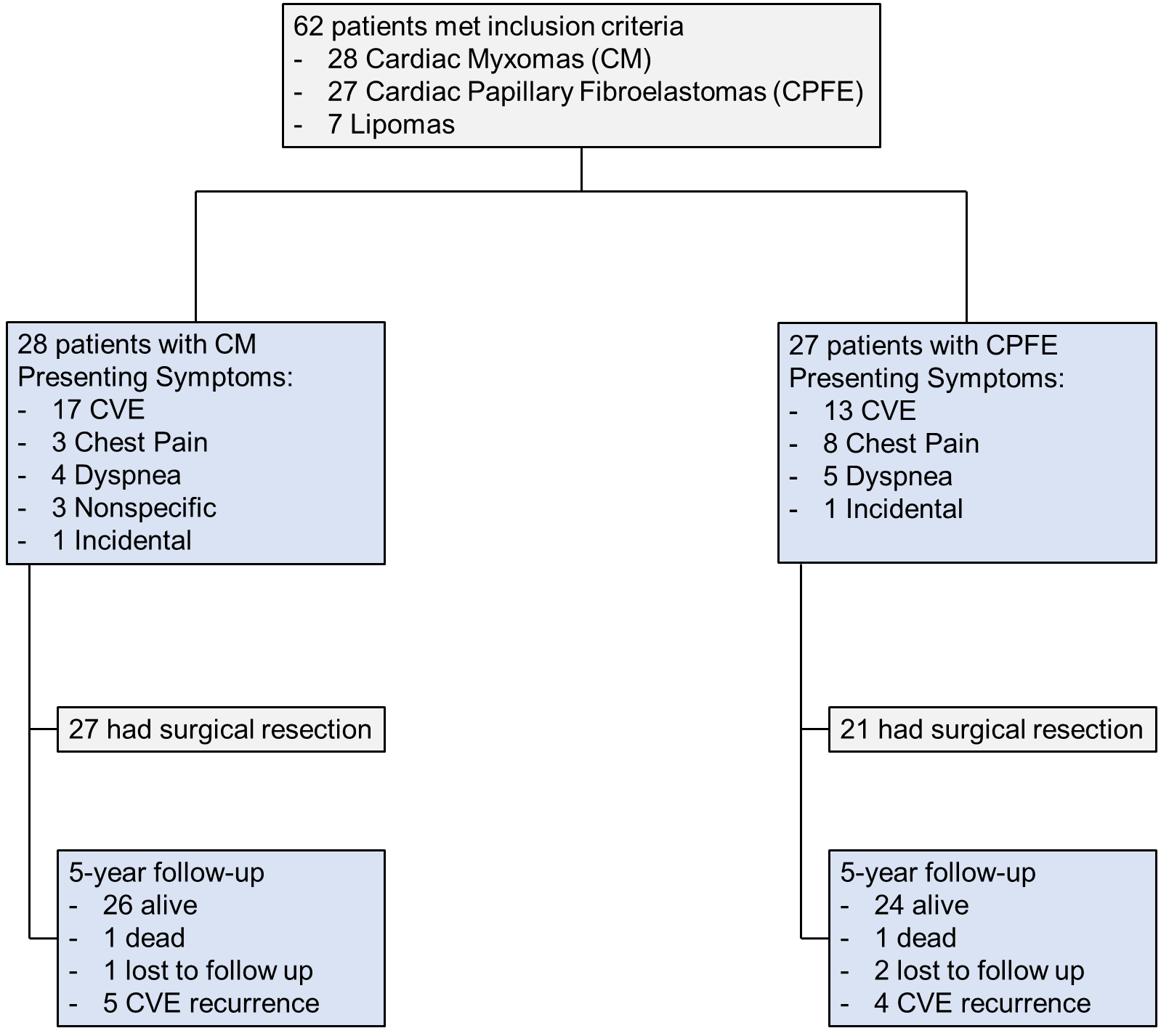


**Figure S1.** Summary of benign cardiac neoplasms investigated in this study, including clinical presentation and follow-up data.

CM= cardiac myxoma; CPFE= cardiac papillary fibroelastoma; CVE= cerebrovascular event.
